# Performance of the pediatric index of mortality 2 (PIM-2) in cardiac and mixed intensive care units in a tertiary children’s referral hospital in Italy

**DOI:** 10.1186/1471-2431-13-100

**Published:** 2013-06-25

**Authors:** Marta Luisa Ciofi degli Atti, Marina Cuttini, Lucilla Ravà, Silvia Rinaldi, Carla Brusco, Paola Cogo, Nicola Pirozzi, Sergio Picardo, Franco Schiavi, Massimiliano Raponi

**Affiliations:** 1 Medical Direction, Bambino Gesù Children’s Hospital, Piazza S. Onofrio, 4, 00161, Rome, Italy; 2 Unit of Epidemiology, Bambino Gesù Children’s Hospital, Rome, Italy; 3 Cardiac Surgery Intensive Care Unit, Bambino Gesù Children’s Hospital, Rome, Italy; 4 Department of Anesthesia and Intensive Care, Bambino Gesù Children’s Hospital, Rome, Italy

**Keywords:** Critical care, Risk adjustment, Mortality pediatric index of mortality, Cardiac surgery, Pediatrics, Quality indicators

## Abstract

**Background:**

Mortality rate of patients admitted to Intensive Care Units is a widely adopted outcome indicator. Because of large case-mix variability, comparisons of mortality rates must be adjusted for the severity of patient illness at admission. The Pediatric Index of Mortality 2 (PIM-2) has been widely adopted as a tool for adjusting mortality rate by patients’ case mix. The objective of this study was to assess the performance of PIM-2 in children admitted to intensive care units after cardiac surgery, other surgery, or for other reasons.

**Methods:**

This was a prospective cohort study, conducted in a 607 inpatient-bed tertiary-care pediatric hospital in Italy, with three pediatric intensive care Units (PICUs) and one cardiac Unit (CICU). In 2009–11, all consecutive admissions to PICUs/CICU of children aged 0–16 years were included in the study. Discrimination and calibration measures were computed to assess PIM-2 performance. Multivariable logistic regression analysis was used to assess the association of patients’ main reason for intensive care admission (cardiac-surgical, other-surgical, medical), age, Unit and year with observed mortality, adjusting for PIM-2 score.

**Results:**

PIM-2 data collection was completed for 91.2% of total PICUs/CICU patient admissions (2912), and for 94.8% of patients who died in PICUs/CICU (129). Overall observed mortality was 4.4% (95% CI, 3.7-5.2), compared to 6.4% (95% CI, 5.5-7.3) expected mortality. Standardised mortality ratio was 0.7 (95% CI: 0.6-0.8). PIM-2 discrimination was fair (area under the curve, 0.79; 95% CI: 0.75-0.83). Calibration was less satisfactory, mainly because of the over two-fold overprediction of deaths in the highest risk group (114.7 vs 53; p < 0.001), and particularly in cardiac-surgical patients. Multivariable logistic analysis showed that risk of death was significantly reduced in cardiac-surgical patients and in those aged 1 month to 12 years, independently from PIM-2.

**Conclusions:**

The children age distribution and the proportion of cardiac-surgical patients should be taken into account when interpreting SMRs estimated using the PIM-2 prediction model in different Units. A new calibration study of PIM-2 score might be needed, and more appropriate cardiac-focused risk-adjustment models should be developed. The role of age on risk of death needs to be further explored.

## Background

The mortality rate of patients admitted to Intensive Care Units is a widely adopted outcome indicator, and in children it was reported to vary from 3.8% to 13% in North and South America and in Europe [1-4]. Because of case-mix variability, comparisons of mortality between different Units, and in the same Unit over time, must be adjusted for the severity of patient illness at admission. To this end, severity-scoring systems specific for the pediatric population have been developed [5].

The Pediatric Index of Mortality 2 (PIM-2) score uses a logistic regression model to obtain an equation that describes the relationship between a limited set of predictor variables measured at the time of admission to intensive care and the probability of death. Originally developed in Australia in the mid-1980’s [6], the score was revised to version 2 in late 1990’s [7] to account for the changes in intensive care organization and outcomes over time. Since then, PIM-2 has been widely adopted as a tool for adjusting mortality rate by patients’ case mix [8-10].

However, its performance has recently been questioned for pediatric cardiac surgery patients, as a study carried out in the United States showed that in this group discrimination was lower than previously reported, and calibration was poor [11]. Whether or not the same result would be obtained in pediatric patients treated in a specialized dedicated cardiac ICU was, according to the Authors [11], an open question.

The Bambino Gesù Children’s Hospital is a 607 inpatient-beds tertiary care and research institute in Rome, Italy, with one pediatric cardiac surgery Intensive Care Unit (CICU) and three multidisciplinary medical/surgical (M/S) pediatric Intensive Care Units (PICUs), two of which participated in a national PIM-2 validation study conducted in years 2004–2005 [10].

This study is aimed at assessing the performance of PIM-2 in children admitted to the three PICUs and to CICU after cardiac surgery, other surgical procedures, or for other reasons.

## Methods

### Setting

The Bambino Gesù Children’s Hospital is the largest pediatric hospital in Italy, and a referral center for pediatric cardiac surgery, pediatric trauma, organs and bone marrow transplants and extracorporeal membrane oxygenation. Annual hospital acute inpatient admissions were 30,344 in 2009, 23,796 in 2010, and 24,449 in 2011. Over this period the average diagnosis-related group weight at hospital discharge, a measure of disease complexity, increased from 0.92 in 2009, to 1 in 2010 and 1.03 in 2011. The average length of hospital stay also increased, being 5.9, 6.3 and 6.4 days from 2009 to 2011.

The Bambino Gesù CICU (14 beds) is a dedicated pediatric cardiac and cardiac surgical Unit. The three M/S PICUs have a total of 23 beds; PICU1 and PICU3 mostly admit surgical patients, including transplants (PICU1), and hospitalised patients who become critically ill; PICU2 covers all the intensive care admission from the Emergency Service and also neurosurgical patients. All Intensive Care Units have their own medical and nursing staff.

The collection and analysis of PIM-2 data for estimating Standardised Mortality ratios (SMRs) in each Intensive Care Unit was one of the objectives of the 2009–2011 annual Hospital Quality Improvement Programs. These objectives are annually reviewed and approved by the Hospital Quality Committee.

### Data collection

All consecutive patient admissions to one of the four hospital PICUs/CICU from 1 January 2009 to 31 December 2011 were included in the study. Preterm babies below 32 weeks of gestational age and/or birth weight < 1500 grams, and adolescents >16 years of age were excluded. Patients re-admissions to PICU/CICU were included as new admissions only if occurred ≥ 48 hours from transfer to another hospital ward; otherwise they were considered as one single admission [10].

Intensive care physicians prospectively collected the data required for PIM-2 calculations [7] within one hour from time of admission to PICU/CICU. Data on patient age, gender, length of intensive care stay, outcome (dead/alive) at discharge from PICU/CICU and ICD9 codes of main diagnosis at discharge from hospital were abstracted from patients’ medical record. The variable “main reason for CICU/PICU admission” was created using information on surgical procedures performed before transfer to intensive care, and patient diagnosis at discharge. It was then categorized in the following three groups: recovery from cardiac surgical procedure (cardiac surgical patients); recovery from other surgery (other surgical patients); other reasons than recovery from surgery (medical patients). Patients still in the PICU at the end of the study were considered alive.

### Data analysis

All analyses were carried out at the level of the individual admission to intensive care (unit of analysis).

Characteristics of patients consecutively admitted to PICU/CICU were reported according to the type of Intensive Care Unit. Differences were tested in univariate analyses using the *χ*2 or Fisher exact tests for categorical variables, and non parametric analysis (Kruskal Wallis test for medians) for continuous variables.

Discrimination and calibration measures were used to assess the PIM-2 performance. Discrimination, i.e. the ability of the PIM-2 score to correctly distinguish between survivors and non-survivors at discharge from ICU, was assessed by computing the Receiver Operating Characteristic (ROC) curve and underlying area (Area Under the Curve; AUC) on the total sample and by Unit, main reason of PICU/CICU admission (i.e., cardiac surgical, other surgical and medical), year and patient age class. Age class was defined using the six-group classification adopted by the International Sepsis Forum [12], i.e., newborn (< 7 days of life); neonate (7–30 days); infant (1–12 months); pre-school (1–5 years); school (6–12 years); adolescent (13–16 years). An AUC > 0.70 was considered to indicate acceptable discriminatory performance [13].

Calibration, or agreement between the predicted and the actual observed number of deaths, was measured using the Hosmer-Lemeshow goodness-of-fit chi-squared test by groups of predicted mortality risk, on the total sample and by main reason of PICU/CICU admission. The Hosmer-Lemeshow test was calculated as Σ (O - E)^2^/E, where O is observed and E is expected (or PIM-2-standardized) number of events in each group of risk [14]. For this test, p value >0.05 indicates good calibration.

Standardised Mortality ratios (SMRs) and 95% confidence intervals (CI) were computed as the number of observed deaths divided by the deaths predicted by the PIM-2 score. SMRs were calculated in the total sample, by Unit, main reason of PICU/CICU admission, year and age class.

A multivariable regression analysis was performed to assess whether other variables could predict the risk of death independently from PIM2 score. In detail, this analysis simultaneously investigated the association between main reason for PICU/CICU admission, age class, year of admission, type of Intensive Care Unit and observed mortality, adjusting for PIM-2 score. The final model retained all the variables significantly associated with mortality at p < 0.05 level.

Data analyses were performed using the STATA statistical package, version 12 (Stata Corp, College Station, TX).

## Results

During the study period, there were a total of 3,194 patient admission to PICUs/CICU. PIM-2 data were completed for 91.2% of admissions (n = 2,912). In 282 admissions PIM2 score could not be computed because of missing values.

CICU contributed to 46.6% of cases (Table 1). Compared to admissions to the three PICUs, CICU admissions involved younger patients (about 23% ≤ 30 days of life). In > 90% of cases they were affected by cardiovascular congenital malformations or cardiovascular diseases; 81% were admitted to CICU after cardiac surgical procedures, while the other cases did not report recovery from cardiac surgery as main reason for CICU admissions. In contrast, PICU3 admissions concerned older patients (about 59% ≥6 years of age), mostly affected by diseases of the musculoskeletal (39.6%) or respiratory (24.3%) systems. Over 77% of them were admitted to PICU after surgical procedures other than cardiac. In the other two PICUs, respiratory diseases were the most frequent main discharge diagnosis (31.4 and 28.2% respectively), followed by cancer (12.8%) and gastro-intestinal disorders (10.2%) in PICU1, and by diseases of the nervous system and sense organs (18.4%), injuries and poisonings (12.9%) in PICU2. In those two PICUs, other surgical and medical PICU admissions were evenly distributed.

**Table 1:** Characteristics of patients admissions to intensive care unit, by type of unit

	**CICU**		**PICU 1**		**PICU 2**		**PICU 3**			**Total**	
	**(N. 1,356)**		**(N. 538)**		**(N. 697)**		**(N. 321)**			**(N. 2,912)**	
	**N**	**%**	**N**	**%**	**N**	**%**	**N**	**%**	**P-value**	**N**	**%**
**Gender**									< 0.001		
Male	743	54.9	300	56.2	436	62.6	137	43.0		1,616	55.7
Female	611	45.1	234	43.8	261	37.5	182	557.0		1,288	44.3
**Age class**									< 0.001		
Newborn (0-7d)	188	13.9	20	3.7	43	6.2	0	0.0		251	8.6
Neonate (8–30 d)	125	9.2	12	2.3	35	5.0	3	0.9		175	6.0
Infant (1–12 m)	409	30.2	141	26.2	214	26.3	49	15.2		813	27.9
Preschool (1–5 y)	378	27.9	204	37.9	256	27.9	79	24.6		917	31.5
School (6–12 y)	170	12.5	112	20.8	81	18.7	70	21.8		433	14.9
Adolescent (13-16y)	86	6.3	49	9.1	68	21.1	120	37.4		323	11.1
**Elective admissions in PICU/CICU**									< 0.001		
No	171	12.6	333	61.9	389	55.8	66	20.6		959	32.9
Yes	1185	87.4	205	38.1	308	44.2	255	79.4		1,953	67.1
**Main reason for PICU/CICU admission**							< 0.001				
Cardiac surgical	1100	81.1	12	2.2	20	2.9	8	2.5		1,140	39.2
Other surgical	106	7.8	248	46.1	349	50.1	249	77.6		952	32.7
Medical	150	11.1	278	51.7	328	47.1	64	19.9		820	28.2
**Length of stay in PICU/CICU** Median (Range)	2 (0.0 – 564.0)		3 (0.0; 220.0)		3 (0.0; 111.0)		1 (0.0 – 365.0)		< 0.001	2 (0.0 – 564.0)	
**Main Diagnosis at Hospital discharge**									< 0.001		
Congenital anomalies and other diseases of the circulatory system	1,229	90.6	41	7.6	34	4.9	9	2.8		1,313	45.1
Diseases of the respiratory system	18	1.3	169	31.4	201	28.2	78	24.3		466	16.0
Diseases of the nervous system of the sense organs	5	0.4	38	7.1	128	18.4	16	5.0		187	6.4
Injury and poisoning	67	4.9	13	2.4	90	12.9	4	1.3		174	5.6
Diseases of the musculoskeletal system and connective tissue	1	0.1	0	0.0	0	0.0	127	39.6		128	4.4
Diseases of the digestive system	4	0.3	55	10.2	23	3.3	28	8.7		110	3.8
Infectious and parasitic diseases	1	0.1	13	2.4	11	1.6	0	0		25	0.9
Congenital anomalies other than circulatory system	8	0.6	56	10.4	54	7.8	33	10.3		151	5.2
Cancer	6	0.4	69	12.8	69	9.9	12	3.7		156	5.4
Other	17	1.3	84	15.6	87	12.5	14	4.4		202	6.9

Overall, 174 patients were readmitted to the same PICU/CICU > 48 hours from being transferred to another hospital ward, accounting for a total number of 387 admissions (13% of the 2,912 admissions considered, data not shown in table). These children experienced a median number of 2 admissions per patient (range: 2–5). The main reason for PICU/CICU admission was cardiac surgical for the majority of cases (169/387, 43.7%), followed by medical (130; 33.6%) and other surgical (88; 22.7%). Median length of stay in PICU/CICUs was 4 days per admission (range: 0–244 days).

In the years 2009–2011, 136 patients died in PICUs/CICU; PIM-2 was collected for 129/136 (94.8%). The seven patients who died and had no PIM-2 data were admitted in PICU/CICU for medical causes (four patients, aged 5, 10, 12 and 13 years, respectively) or for other surgical procedures (three patients aged 11, 14 and 15 years respectively).

Table 2 shows the observed and PIM-2-standardized (expected) mortality rates by Unit type, patients’ characteristics, and year. Overall the observed mortality was 4.4% (95% CI, 3.7-5.2), but varied significantly according to reason for intensive care admission (p < 0.001), and patient age class (p < 0.001).

**Table 2:** Model fit and discrimination by type of unit, main reason for PICU/CICU admission, year and age class

	**Observed mortality**			**Expected mortality**			**SMR**		**ROC curve**	
				**(PIM-2 standardized)**						
	**N**	**%**	**95% CI**	**N**	**%**	**95% CI**	**SMR**	**95% CI**	**AUC**	**95% CI**
**Unit**										
CICU	54	4.0	(3.0 - 5.2)	88.8	6.6	(5.3 - 8.0)	0.6	(0.5 - 0.8) ^§^	0.72	(0.65 - 0.80)
PICU1	29	5.4	(3.6 - 7.6)	49.1	9.1	(6.8 - 11.9)	0.6	(0.4 - 0.9) ^#^	0.76	(0.66 - 0.85)
PICU2	38	5.5	(3.9 - 7.4)	35.6	5.5	(3.6 - 7.1)	1.1	(0.8 - 1.5)	0.85	(0.78 - 0.92)
PICU3	8	2.5	(1.1 - 4.9)	13.0	4.0	(2.2 - 6.8)	0.6	(0.3 - 1.2)	0.91	(0.80 - 1.00)
**Main reason for PICU/CICU admission**										
Cardiac surgical	37	3.3	(2.3 – 4.4)	73.0	6.4	(5.1 – 8.0)	0.5	(0.4 – 0.7) ^§^	0.72	(0.63 – 0.82)
Other surgical	28	2.9	(2.0 – 4.2)	26.4	2.7	(1.8 – 4.0)	1.1	(0.7 – 1.5)	0.85	(0.77 – 0.93)
Medical	64	7.8	(6.1 – 9.9)	87.0	10.6	(8.6 – 12.9)	0.7	(0.6 – 0.9) *	0.76	(0.69 – 0.82)
**Year**										
2009	48	4.7	(3.5 - 6.2)	69.1	6.7	(5.3 - 8.5)	0.7	(0.5 - 0.9) ^#^	0.81	(0.74 - 0.88)
2010	47	4.4	(3.3 - 5.8)	65.3	6.1	(4.7 - 7.7)	0.8	(0.5 - 0.9) *	0.82	(0.76 - 0.88)
2011	34	4.1	(2.9 - 5.7)	51.9	6.3	(4.8 - 8.2)	0.7	(0.4 - 0.9) *	0.73	(0.63 - 0.80)
**Age class**										
Newborn (0-7d)	26	10.4	(6.9 - 14.8)	20.3	8.0	(4.9 - 12.0)	1.3	(0.8 - 1.9)	0.74	(0.66 - 0.83)
Neonate (8-30d)	11	6.3	(3.2 - 11.0)	14.1	8.0	(4.4 - 13.1)	0.8	(0.4 - 1.4)	0.79	(0.66 - 0.92)
Infant (1-12 m)	31	3.8	(2.6 - 5.4)	49.7	6.1	(4.6 - 8.0)	0.6	(0.4 - 0.9) ^#^	0.69	(0.58 - 0.80)
Preschool (1-5y)	26	2.8	(1.9 - 4.1)	55.3	6.0	(4.5 - 7.7)	0.5	(0.3 - 0.7) ^§^	0.84	(0.74 - 0.93)
School (6-12y)	15	3.5	(2.0 - 5.6)	26.8	6.2	(4.1 - 8.9)	0.6	(0.3 - 0.9) *	0.84	(0.76 - 0.92)
Adolescent (13-16y)	20	6.2	(3.8 - 9.4)	20.2	6.2	(3.8 - 9.4)	1.0	(0.6 - 1.5)	0.87	(0.79 - 0.95)
**Total**	129	4.4	(3.7 - 5.2)	186.4	6.4	(5.5 - 7.3)	0.7	(0.6 - 0.8) ^§^	0.79	(0.75 - 0.83)

*: observed versus expected deaths; p < 0.05.

^#^: observed versus expected deaths; p < 0.01.

^§^: observed versus expected deaths; p < 0.001.

The total number of expected deaths was 186.4, accounting for a PIM-2 predicted mortality risk of 6.4% (95% CI, 5.5-7.3) and SMR of 0.7 (95% CI, 0.6-0.8). The number of observed deaths was significantly lower than expected in CICU and PICU1, as indicated by SMR confidence limits both below 1. The SMR was < 1 also in PICU3 (0.6), but this result was not statistically significant (95% CI: 0.3-1.2).

Compared to expected, observed mortality was significantly lower also in cardiac surgical and medical admissions, while numbers of observed and expected deaths were very close in the other surgical group (28 vs 26.4; SMR 1.1). Mortality rates and SMRs remained stable over the three years of the study.

**Figure 1:**
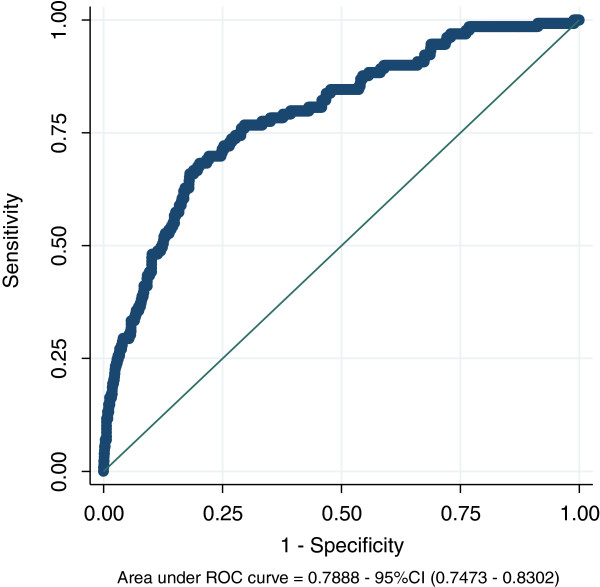
Receiving operating characteristic curve; entire cohort.

Overall, the value of the area under the curve (AUC) was 0.79 (95% CI, 0.75-0.83), indicating good discrimination ability. The full ROC curve for the total sample is shown in Figure 1. The discrimination ability was over 0.70 for all reasons of admission to intensive care, though it was lowest among the cardiac surgical and highest among the other surgical patients (Table 2).

The Hosmer-Lemeshow test showed a suboptimal overall goodness of fit (129 observed versus 186.4 expected deaths; p < 0.001) (Table 3). This result was mainly due to the overprediction of deaths in the highest risk centile (53 versus 114.7, SMR 0.5, 95% CI 0.4-0.6). Overprediction of deaths in the highest risk group was confirmed for cardiac surgical (11 observed vs 43.8 expected deaths) and medical patients (27 observed vs 45.4 expected deaths), but was not present in the other surgical patients (results not shown in table).

**Table 3:** Hosmer-Lemeshow goodness of fit test for groups of mortality risk; entire cohort

**ALL (HL chi2 **[10] **= 68.0; p < 0.001)**									
**Group of risk**	**N**	**Obs**	**%**	**Exp**	**%**	**HL**	**SMR**	**95% CI**	**P-value**
0.000 - 0.003	291	2	(0.7)	0.6	(0.2)	3.2	3.3	(0.4 - 11.9)	NS
0.003 - 0.006	291	0	(0.0)	1.2	(0.4)	1.2	0.0	(0.0 - 3.0)	NS
0.006 - 0.010	291	5	(1.7)	2.2	(0.8)	3.4	2.2	(0.7 - 5.2)	NS
0.010 - 0.014	291	7	(2.4)	3.4	(1.2)	4.0	2.1	(0.8 - 4.3)	NS
0.014 - 0.018	292	6	(2.1)	4.6	(1.6)	0.4	1.3	(0.5 - 2.9)	NS
0.018 - 0.025	291	7	(2.4)	6.1	(2.1)	0.1	1.2	(0.5 - 2.4)	NS
0.025 - 0.035	291	6	(2.1)	8.5	(2.9)	0.8	0.7	(0.3 - 1.5)	NS
0.035 - 0.066	291	13	(4.5)	14.1	(4.9)	0.1	0.9	(0.5 - 1.6)	NS
0.066 - 0.175	291	30	(10.3)	30.9	(10.6)	0.0	1.0	(0.7 - 1.4)	NS
0.175 - 1.000	292	53	(18.2)	114.7	(39.3)	54.7	0.5	(0.4 - 0.6)	< 0.001
**Total**	2,912	129	(4.4)	186.4	(6.4)	68.0	0.7	(0.6 - 0.8)	< 0.001

The multivariable logistic analysis showed that, independently from PIM-2, main reason of PICU/CICU admission and patients’ age remained significantly associated with observed mortality, while year and Unit were not (Table 4). Compared to medical patients, cardiac surgical patients had a significantly lower risk of deaths. Babies in the first month of life and adolescents had a significantly increased risk of death compared to children from 1 month to 12 years of age.

**Table 4:** Logistic multivariable analysis of factors associated with risk of death

	**OR**	**95% CI**	**P-value**
**PIM-2 score**	53.2*	(25.2- 112.7)	< 0.001
**Main reason for PICU/CICU admission**			0.009
Medical	Ref.	-	
Other surgical	0.7	(0.4 – 1.1)	
Cardiac surgical	0.5	(0.3 – 0.8)	
**Age class**			< 0.001
Newborn (0-7d)	3.7	(2.0 – 6.8)	
Neonate (8-30d)	2.3	(1.0 – 5.0)	
Infant (1-12 m)	1.5	(0.9 – 2.6)	
Preschool (1-5y)	Ref.	-	
School (6-12y)	1.3	(0.7 – 2.5)	
Adolescent (13-16y)	2.2	(1.2 – 4.2)	

* for each incremental point of PIM-2 value.

Ref: reference group.

## Discussion

We studied the performance of PIM-2 in a large sample of patients admitted during 2009–2011 to three PICUs and one CICU of a large tertiary pediatric hospital in Italy. The high rates of completion achieved confirm that the PIM-2 score is easy to apply and includes variables easily available at admission to intensive care. PIM-2 appeared to have a fair discriminating ability, with an overall AUC of 0.8. However, calibration as assessed by Hosmer-Lemeshow goodness of fit test was less satisfactory, with statistically significant overprediction of deaths in the total sample, mainly due to the over two-fold overprediction in the highest risk group.

Similar findings were reported by other Authors [11,15-17], and variably attributed to differences in the characteristics of the study population, small sample size, particularly for number of deaths, and improvement in the quality of intensive care making the original PIM-2 equation, developed about ten years ago, no longer fully appropriate.

Poor calibration by risk groups was not observed in the multicentric study validating PIM-2 for the Italian population [10]. This validation study was conducted in 2004–2005, and we cannot exclude that quality of care has further improved since then. Additionally, that study included 18 between pediatric and general hospitals with a pediatric ICU, and Unit organization and care delivered are likely to have been less homogeneous in a such large sample of different hospitals than between the ICUs of a single hospital entirely dedicated to the care of children.

In our study, overprediction of deaths was however particularly evident among cardiac surgical admissions, where the number of expected deaths in the highest risk group was approximately four times higher than observed. These results are in agreement with those found by Czaja et al. in their study of cardiac surgical pediatric patients in 55 US PICU in 2005–07 [11]. The mortality reported by this study (3.4%) was very close to the 3.3% rate observed in our population of children who underwent cardiac procedures. Also discrimination was similar in the two studies, with AUC values of 0.72 (CI 0.63-0.82) in Italy, and 0.80 (95% CI, 0.77– 0.83) in US, while standardised mortality rates were 0.5 (CI 0.4-0.7) and 0.8 (0.7–0.9), respectively.

In our hospital cardiac and cardiac surgical patients are treated in a separate intensive care Unit (CICU), characterized by highly specialized dedicated staff and high activity volume. Consistency between our results and those by Czaja et al. [11] suggests that PIM-2 calibration for cardiac surgical patients may indeed be sub-optimal. The development of a specific algorithm for predicting the risk of death in this highly selected category of patients would thus be helpful.

At the light of the statistically significant differences in observed mortality found between Units, main reasons of admission to intensive care and patient age class, we performed a multivariable analysis to simultaneously explore the effect of these variables on risk of death, adjusting for PIM2 score. In fact, SMRs did vary by Unit, and different Units admitted patients with different age distribution and main reason for admission. It was then of interest to investigate whether in our patient population these variables and the Unit of admission may have a significant influence on mortality in excess to the proportion predicted by PIM-2 score.

We found that, independently from PIM-2, main reason for admission and age remain significant predictors of the observed mortality, with lower risk in cardiac surgical patients and in children from 1 month to 12 years of age, and higher risk in medical patients and in babies < 1 month of age or adolescents. It is interesting to note that the two Units with SMRs significantly lower than 1 admitted mostly patients in the cardiac surgical group (CICU) or from 1 month to 12 years of age (PICU1), confirming the importance of differences of patient case-mix.

The lower risk in cardiac surgical patients supports the importance of cardiac focused risk adjustment. The role of age on risk of death needs to be further explored. In our study, the observed mortality was higher in the first month of life and in adolescents. In these age groups, the PIM-2 standardized mortality was very close to the observed mortality, while it was significantly lower in children from 1 month to 12 years of age. The highest risk of dying in newborns, neonates and adolescents observed in multivariate analysis could be due to differences in outcome independent from patients severity at PICU/CICU admission. In fact, both patients < 1 month of age and adolescents have specific characteristics, and could require a more targeted care. Babies < 1 month of age requiring intensive care can also be treated in neonatal Intensive Care Units (NICU). It would thus be relevant to compare PIM-2 performance and SMRs in newborns and neonates admitted in NICUs versus PICUs. Teenagers are different from both adults and pre-adolescent children and, as such, require special attention in the manner in which healthcare services are provided. However, there are no standards to guide the model of care that should be provided specifically for teenagers in intensive care [18].

Potential limitations of this study should be taken into account. Data regarding the main diagnosis were derived from patients’ medical record at discharge, and misclassification cannot be completely excluded. However, in our hospital all clinical records are independently reviewed at discharge by a second physician, who verifies the appropriateness of diagnoses and procedures coding. We derived main reason for PICU/CICU admission from routinely collected data, and more detailed information on reason for intensive care admission was not available. Data related to PIM-2 were collected prospectively by intensive care physicians at time of admission, and rates of completeness were very high both on total patients (91%) and deaths (95%).

## Conclusions

In our study, PIM-2 appeared to have a fair discriminating ability. As other Authors have reported, however, calibration of the score was suboptimal, mainly because of the over two-fold overprediction of deaths in the highest risk group (114.7 vs 53; p < 0.001), and particularly in cardiac-surgical patients. A new calibration study of PIM-2 score might be needed to take into account improvements in quality of care occurred during the last decade. More appropriate cardiac-focused risk-adjustment models for these patients should also be developed. Given the independent role of main reason of admission and age on mortality risk, the proportion of cardiac surgical patients and patient age distribution should be considered in interpreting SMRs estimated by different Units by applying the PIM-2 prediction model, as variable case-mix could distort the overall results and bias comparisons [19].

## Competing interests

The authors declare that they have no competing interest.

## Authors’ contributions

MCDA contributed to the conception of study and interpretation, and writing the manuscript. MC participated in the statistical analysis, interpretation, and writing the manuscript. LR conducted the statistical analysis. SR and CB participated in the design and data collection. PC, NP, SP and FS participated in the data collection and interpretation. MR participated in the design and interpretation. All authors read and approved.

## Pre-publication history

The pre-publication history for this paper can be accessed here:

http://www.biomedcentral.com/1471-2431/13/100/prepub
